# Effect of needle bevel type on pain perception in children during inferior alveolar nerve block anesthesia: randomized controlled clinical trial

**DOI:** 10.1186/s12903-025-06731-7

**Published:** 2025-09-02

**Authors:** Rasha H. Eleraky, Mona Mohy ElDin, Yomna Ibrahim, Reham S. Soliman, Nadia A. Wahba

**Affiliations:** 1 Pediatric Dentistry and Dental Public Health Department, Faculty of Dentistry, Alexandria University, 32 Champollion St. Azarita, Alexandria, 21525 Egypt; 2 Dental Biomaterials Department, Faculty of Dentistry, Alexandria University, Alexandria, Egypt

**Keywords:** IANB, Anesthesia, Needle bevel, Needle deformation

## Abstract

**Background:**

The difficulty in providing optimal treatment has been related to patients’ fear of the discomfort that comes with anesthetic needle insertion. The main purpose of this study is to clinically assess the effect of needle bevel on patient’s perception of pain during inferior alveolar nerve block (IANB) anesthesia and evaluate the needle tip for mechanical deformation.

**Materials and methods:**

Sixty-six children 5–7 years old, who were indicated for IANB anesthesia, were selected from the outpatient clinic of Pediatric Dentistry and Dental Public Health Department, Faculty of Dentistry, Alexandria University. Participants were randomly allocated into two groups according to the type of needle bevel used. Group I (*n* = 33) received INAB anesthesia using double scalpel blade bevel needle, while group II (*n* = 33) received the injection using conventional bevel needle. Pain reaction was evaluated subjectively using modified Maunuksela face pain scale (FPS) and objectively using Sensory, Eye, and Motor (SEM) scale. After the respective injections, the needles were prepared for microscopic examination.

**Results:**

The FPS and SEM showed significantly lower pain scores during IANB anesthesia with double scalpel blade bevel needle compared to the conventional bevel needle (*p* = 0.002, *p* = 0.003 respectively). No significant difference in mechanical angle deformation between the two needles was observed (*p* = 0.837).

**Conclusion:**

For IANB anesthesia, double scalpel blade bevel needle demonstrated significantly less injection pain. All dentalneedles demonstrated tip deformation after single use.

**Supplementary Information:**

The online version contains supplementary material available at 10.1186/s12903-025-06731-7.

## Background

Child management is a routine primary challenge facing pediatric dentists. Since its foundation, the primary aim of dental medicine was to create a painless experience for the patients [1]. The main challenge in providing dental care has always been pain, and fear associated with needle injection [2–4]. Patient management techniques have ever since been in continuous development in an attempt to create a more comfortable local anesthetic injection experience. These include -but are not confined to- smaller gauge needle sizes, slow computer regulated injection, topical agents [5], pre-cooling of injection, vibration using modern devices [6], warming, buffering [7], distraction techniques and adjusting the rate of local anesthesia infiltration [8].

Limited studies in the literature, linking the fact that the size and design of the dental needle seem to affect pain experience during injection [1, 9]. However, few others concluded a possible association between bevel design and the patient’s pain experience [10–12]. Steele et al. [13] performed an in vitro study concluding that an asymmetrical bevel requires lower penetration force than a standard needle. Furthermore, Omoigui et al. [14] reinforced a possible relationship between bevel design and pain upon injection. The limitation of the latter study was the limited number of patients.

Needle bevel design affects needle tip deformation after local anesthetic infiltration, creating barbed hooks on the cutting edge which may result in greater soft tissue trauma and consequently greater pain experience during needle withdrawal [15].

Inferior alveolar nerve block (IANB) is the most commonly used technique for local anesthesia when performing restorative and surgical procedures in the mandible [16]. It is a routine local anesthetic procedure and is generally considered very safe [17]. Since it is an invasive technique, it has lots of inherent potential risks for the patients. Complications may include pain, burning sensation, penetration of a blood vessel, edema, hematoma, trismus, nerve damage, facial nerve paralysis, needle breakage and adverse drug reactions such as overdose, allergy, or idiosyncrasy [18, 19]. IANB is considered one of the most painful techniques of local anesthesia administration, especially in children [18]. This fact is of major concern for the pediatric dentists.

Recently, a patented scalpel-like bevel needle has been introduced [20]. It is claimed to anesthetize the gingiva and pass through cortical bone easily and safely. These needles possess a double scalpel blade bevel which incises the tissue without tearing. The patient no longer feels the prick as the tissues are no longer torn, just incised for a probable atraumatic and painless needle penetration.

Few reports address the possible association between needle bevel design and the patient’s pain perception. Hence, the aim of this study was to compare the difference in pain perception, when using double scalpel blade bevel needle versus standard blade bevel needle during IANB injection and the associated mechanical deformation of the needle tip after the respective single injection. The tested null hypothesis stated that there would be no significant difference neither in pain perception nor in needle deformation when using a double scalpel blade bevel or a standard blade bevel needle during IANB anesthesia injection in children.

## Materials and methods

### Study design

This two-arm, randomized controlled clinical trial ‘‘parallel design’’ was approved by the Research Ethical Committee of the Faculty of Dentistry, Alexandria University, Alexandria, Egypt (IRB NO: 0726072023. IORG0008839). Recruitment of participants and data collection were conducted during February and March 2024. Informed consent from a parent and/or legal guardian of children to participate in the study was also secured. The trial was registered at ClinicalTrials.gov (NCT06242743) 5th February 2024, (retrospective registration). It was conducted and reported following the CONSORT guidelines ‘‘Additional file 1’’ [21], with an allocation ratio 1:1.

### Sample size calculation

Sample size was calculated assuming 5% alpha error and 80% study power. The mean ± SD pain scores were 2.1 ± 1.2 for scalpel designed bevel needle [15] while those for the conventional needle were 3.21 ± 1.89 [22]. Based on the difference between independent means, a sample of 33 patients per group was required, yielding an effect size of the 0.701. Total sample = Number per group x Number of groups = 33 × 2 = 66 patients. Sample size was based on Rosner’s method [23] calculated by G*Power 3.1.9.7 [24].

### Patient recruitment

The study was conducted on sixty-six cooperative children, aged 5- 7years, with a behavioral rating of 3 or 4 according to the Frankl Rating Scale [25]. They were chosen among those attending the outpatient clinic of the Pediatric Dentistry and Dental Public Health Department, Faculty of Dentistry, Alexandria University, Alexandria, Egypt. They were normal and healthy, without systemic disease (rated I, II) according to American Society of Anesthesiologists (ASA) [26]. They all required inferior alveolar nerve block anesthetic injection for dental treatment. Children with hypersensitivity to local anesthetic drugs or those with emergency treatment needs, such as abscess, cellulitis and space infections were excluded from the study. One hundred patients were screened. Sixty-six met the inclusion criteria and participated in the clinical trial. Informed consents from the parents and/or legal guardians to participate in the study were obtained. Patients and their parents were told that the procedure will be videotaped to document their behavior.

### Randomization and allocation concealment

Children chosen to participate in the study were randomly assigned to one of two groups using random allocation. Randomization was done by the permuted block method, and the allocation sequence was generated using random allocation software where participants were allotted in blocks of 6. This was performed using a computer-generated list of random numbers. Each child was given a serial number that was written on identical sheets of paper denoting the group to which each child was allocated and placed inside opaque envelopes containing the respective names of the children [27]. A trial independent personnel was assigned the role of keeping the envelopes and unfolding them only at the time of the local anesthesia injection session.

Each child was assigned to one of the two tested groups: Group I included 33 children who received IANB injection using double scalpel blade bevel needles, while Group II represented the remaining 33 children receiving IANB injection using conventional bevel design [28].

Due to the nature of the study, the operator was not blinded to the treatment groups. However, both patients and the statistician were blinded to the type of needle used.

Intra-examiner reliability was tested by watching videotapes of a group of 10 patients twice with 7days interval between the first and second views. The intra-examiner reliability was tested using Weighted Kappa Coefficient [29]. K was equal 0.808 for assessment of children behavior using (SEM). These patients were not involved in the clinical trial.

Inter-examiner reliability was also assessed by comparing the scores assigned by the outcome assessor with those of the gold standard examiner. Cohen’s kappa value here was 0.73 demonstrating substantial to almost perfect agreement.

A total of ten needles from each group—which were not included in the study sample- were tested under the stereoscopic microscope to determine needle deformation due to error in manufacturing, prior to injection. These served as pilot reference and were immediately discarded after microscopic examination.

### Intervention

Each child was acquainted to the dental setting [30]. The injection site was dried with a gauze, and topical anesthetic gel (Dharma Research, Inc.) was applied for one minute using a cotton applicator. IANB injections were done according to the conventional Halstead technique where the anatomical landmarks were the pterygomandibular raphe, coronoid notch (which are proportionally smaller and less prominent in children) and the occlusal surface [31]. By placing the syringe barrel over the contralateral primary molars, the needle was advanced at the level of the occlusal plane until touching bone to deposit the anesthetic solution near the mandibular foramen [32]. Due to less dense pediatric bones, more gentle insertion and less depth to approximately 15–20 mm, compared to the standard used in adults was adopted [33].

After aspiration, 1 ml of the anesthetic solution was slowly deposited. The volume of anesthetic administered did not exceed the recommended dose according to the patient’s body weight, which is a maximum dose of 7mg/kg [33]. This was to ensure safety and efficacy while minimizing the risk of toxicity. Children weighing between 15 and 30 kg received half a cartridge, while those weighing 30 kg or more were given a full cartridge [34].

All injections were performed by the same operator. A video camera was mounted to record each child’s SEM score during injection.

#### Group I (Test group)

A standard IANB anesthesia (ARTINIBSA, Inibsa Dental S.L.U) was administered using a 27-guage double scalpel blade bevel needle (Effitec*®* Needles, Dental Hi-tec, Cholet, France), mounted on a dental syringe (Beehive Solutions Ltd) at a rate of deposition of 1 ml/60 s. The disposable needle was directed at the level of the occlusal plane from the opposite side until bony resistance was reached. The needle was then withdrawn 2 mm to aspirate. The amount of anesthetic solution administered was calculated according to the patient body weight [34].

#### Group II (Control group)

IANB anesthesia (ARTINIBSA, Inibsa Dental S.L.U) was administered following the same technique described earlier, using the 27-gauge long anesthetic standard blade bevel needles (C-K Ject, CK Dental Ind. Co.).

After each respective single injection, dental needles were collected and covered with caution to avoid deformation [35]. The needles were fixed to an object slide and prepared for microscopic examination. The images were obtained and processed with the computer software Photoshop [15].

All required dental procedures were performed according to the American Academy of Pediatric Dentistry guidelines [36].

### Outcomes assessment

#### Pain

**Fig. 1:**
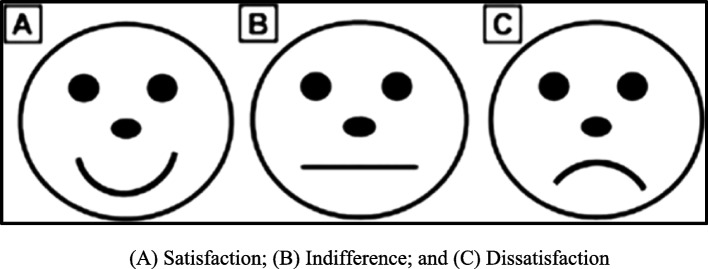
Faces scale modified from the Maunuksela et al. scale

Pain experienced during IANB injection was measured subjectively using the ‘FPS’ modified from Maunuksela et al. [37] (Fig. 1). After injection, each child was requested to select the face that best expressed his/her experience towards the injection.

Objective evaluation was performed by the ‘SEM’ scale [38] to quantify the child’s pain response (Table 1). The observer evaluated each of sounds, eye and body movements during needle injection using recorded video tapes and gave a grade of 1–4. Scores were estimated by adding the three scores and calculating their mean average.

**Table 1:** SEM scale for assessment of children’s behavior

Parameter	Comfort	Mild discomfort	Moderate discomfort	Severe discomfort
Grade	1	2	3	4
Sound	No sound	Non-specific sound (probable pain)	Verbal complaint, louder sound	Verbal complaint shouting, crying
Eye	No sign	Dilated eye without tears (anxiety sign)	Tears, sudden eye movements	Crying, tears all over the face
Motor	Relaxed body and hand status	Muscular contraction, contraction of hands	Sudden body and hand movement	Hand movements for defense, turning the head to the opposite side

#### Needle deformation

The collected needle tips were evaluated under the stereomicroscope (B061, Olympus, Japan) at magnification of × 60 to determine the direction of deviation and measure the area as well as the angle of deformation [35]. Each needle tip was adapted on the central square of a haemocytometer slide (Marienfeld Superior, Germany) on the stereomicroscope stage, where the needle hub was fixed using a customized mold fabricated from the putty consistency of condensation silicone rubber base impression material (Zhermack, Italy). The direction of deviation was determined as either outward deviation (deviation away from needle bevel) or inward (deviation towards needle bevel) [15]. Images of the needle tip were captured then inserted in Photoshop CC software (Adobe Systems Incorporated, USA) in order to measure the area of needle tip deformation arbitrarily by counting the boxes occupied by the deformed portion of the needle tip in a 50 µm2 box grid [15]. The actual deviation area was calculated using Image J software (1.52a, National Institute of Health, USA) by tracing the deviated portion of the needle tip using the freehand selection tool and calculating the area in micro-meter squared. The angle of deviation was also measured by the angle tool in Image J software by applying a straight line to the needle shaft and drawing another line according to the deviation. Angles less than 90º indicated a severe deviation and bending while angles more than 90º and closer to 180º indicated a mild degree of deviation [15]. Needles that were used for pilot evaluation were tested in the same method without being used for injection and were discarded immediately after examination.

### Statistical analysis

Normality was checked using Shapiro Wilk test and Q-Q plots. Non-normal distribution was confirmed for SEM while Age was normally distributed. Data were presented using mean and standard deviation in addition to median, inter quartile range (IQR), minimum, and maximum. Independent t test and Mann Whitney U test were used to compare age, SEM, needle measurements between groups, respectively. Face Pain Scale and needle deviation were analyzed using Chi Square test. All tests were two tailed and the significance level was set at* p* value ≤ 0.05. Data were analyzed using IBM SPSS, version 23 for Windows, Armonk, NY. USA.

## Results

**Fig. 2:**
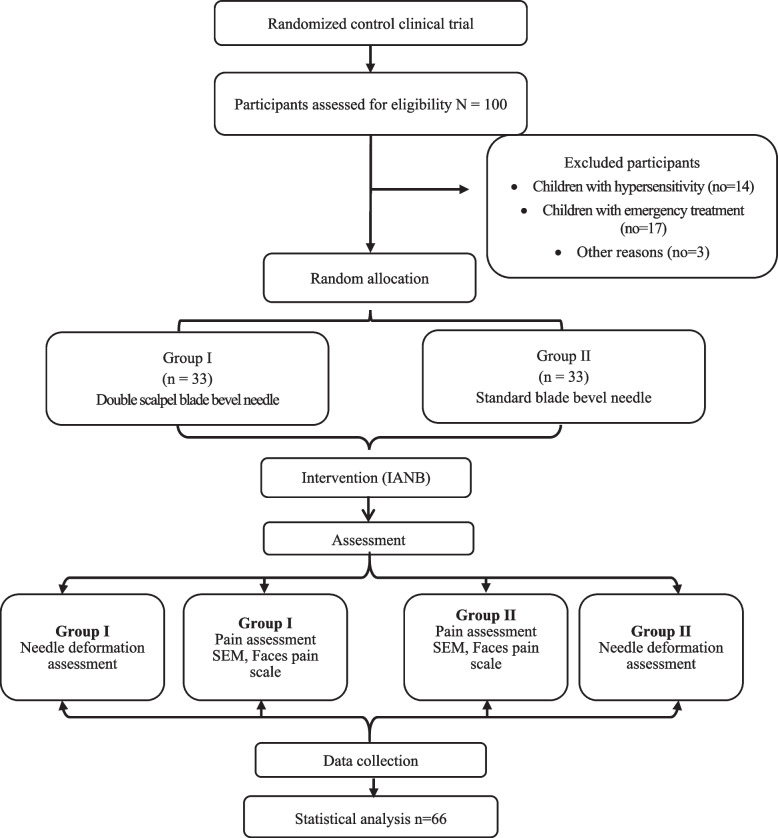
COSNORT flow diagram of participants throughout the study

One hundred children were assessed for eligibility. Sixty-six children met the eligibility criteria and were included in the study. Patients were randomly allocated equally to two groups receiving IANB injections; one using the double scalpel blade bevel needle and the other using the conventional needle (Fig. 2). Mean age of children of the test group was 6.09 ± 0.91 comprising 15 boys and 18 girls while the control group included 18 boys and 15 girls with a mean age of 5.94 ± 0.80. Age and gender were comparable between the two groups with no significant difference (*p*˃0.05). ‘‘Additional file 2’’.

Table 2 shows the comparison between test and control groups regarding FPS expressed in terms of patient satisfaction. Shapiro Wilk test and Q-Q plots were used for quantitative non-normally distributed and ordinal variables. “Satisfaction” was reported in 42.4% of group I patients (double scalpel blade bevel needle), in comparison to only 15.2% who reported the same reaction in group II (conventional needle). There was a statistically significant lower score in perceived pain with double scalpel blade bevel needle compared to conventional one where *p* = 0.002.

**Table 2:** Comparison of FPS between the test and control group

	**Group I (test)** **(*n* = 33)**	**Group II (control)** **(*n* = 33)**	***p* value**
	**N (%)**		
**A (Satisfaction)**	14 (42.4%)	5 (15.2%)	0.002^*^
**B (Indifference)**	15 (45.5%)	11 (33.3%)	
**C (Dissatisfaction)**	4 (12.1%)	17 (51.5%)	

^*^Statistically significant at *p* value ≤ 0.05

**Fig. 3:**
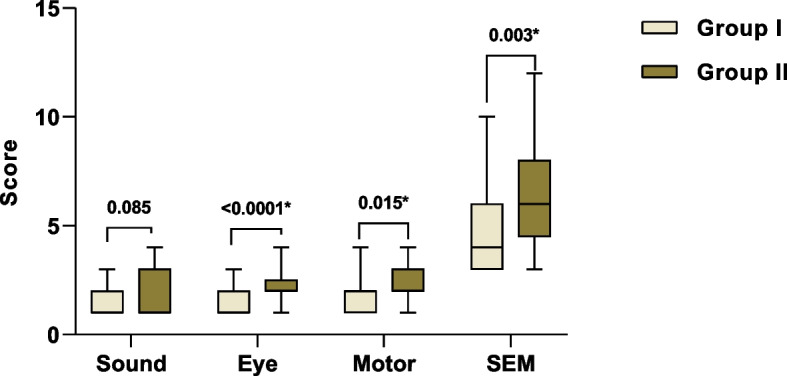
Comparison between the median of Sound, Eye, Motor and total SEM score between Group I (test) and Group II (control)

Figure 3 illustrates a comparison between the medians of Sound, Eye, Motor and overall SEM score. Normality tests showed that the data here was not normally distributed. Significant lower scores regarding the eyes and motor were reported when patients received IANB using the double scalpel blade bevel needle in comparison with conventional needle (*p* < 0.0001, *p* = 0.015 respectively). The median (IQR) of the SEM score with double scalpel blade bevel needle was 4.00 (3.00) which was significantly lower than that of conventional needle where the median (IQR) was 6.00 (4.00), *p* = 0.003.

**Fig. 4:**
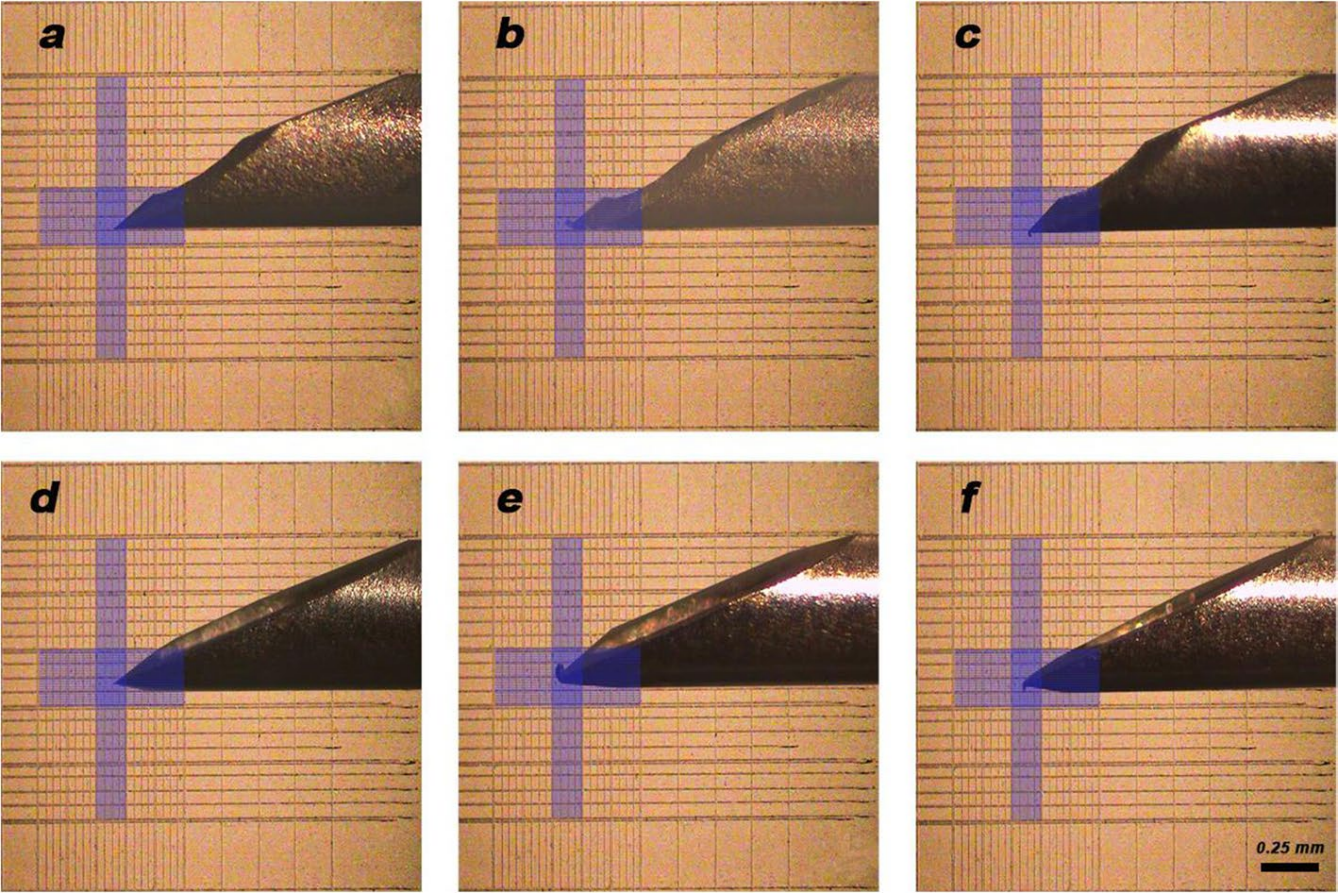
Needle deviation stereomicroscope images (× 60) on grid showing control group (**a-c**) and test group (**d-f**). **a** and **d** no deviation, **b** and **e** inward deviation, and **c** and **f** outward deviation

Figure 4 and Table 3 show a comparison between test and control groups regarding needle deviation and its direction. There was no significant association between the directions of the deviation itself whether inward or outward *p* = 0.867. Meanwhile, (Table 4) shows the changes in needle measurements after use between the test and control groups displaying no significant differences regarding area or angle.

**Table 3:** Comparison between needles’ deviation after use in the two groups

	**Group I** **(*n* = 33)**	**Group II** **(*n* = 33)**	***p* value**
	n (%)		
**No change**	5 (15.2%)	5 (15.2%)	0.867
**Inward**	15 (45.5%)	13 (39.4%)	
**Outward**	13 (39.4%)	15 (45.5%)

**Table 4:** Changes in needles measurements after use between the test and control group

		**Group I (test)** **(*n* = 33)**	**Group II (control)** **(*n* = 33)**	***p* value**
Area/boxes	Mean ± SD	7.85 ± 9.11	15.91 ± 20.42	0.153
	Median (IQR)	6.00 (6.00)	9.00 (16.00)	
	Min—Max	0.00 – 40.00	0.00 – 83.00	
Area/mm2	Mean ± SD	204.58 ± 340.62	612.27 ± 877.84	0.227
	Median (IQR)	94.06 (176.70)	135.23 (1078.44)	
	Min—Max	0.00 – 1584.13	0.00 – 2917.12	
Angle	Mean ± SD	132.03 ± 57.38	116.60 ± 64.11	0.837
	Median (IQR)	152.77 (12.84)	145.70 (115.16)	
	Min—Max	0.00 – 189.20	0.00 – 174.49	

The distribution of satisfaction scores between the two groups showed a statistically significant difference (*p* = 0.002), indicating higher satisfaction levels in Group I compared to Group II. In Group I, 42.9% of participants reported being satisfied (Score A), while only 17.9% in Group II expressed satisfaction. Indifference (Score B) was reported by 46.4% in Group I and 28.6% in Group II. Notably, dissatisfaction (Score C) was significantly more common in Group II (53.6%) compared to Group I (10.7%). According to the correlation analysis, a weak negative relationship was observed between needle angle and anxiety scores in both groups. For Group I, the correlation coefficient was *ρ* = −0.252 (*p* = 0.156), and for Group II, *ρ* = −0.238 (*p* = 0.182) ‘‘Additional file 3’’.

## Discussion

The present study aimed to compare the pain elicited by double scalpel blade bevel needles and conventional needles during IANB injection in children. IANB technique was chosen over infiltration techniques in this study as it is considered one of the most painful injections, so the demand for a less painful experience was critical, particularly for children [39]. The conventional Halstead technique has been the IANB procedure of choice in this study because it has been proven to be one of the simplest compared to other techniques [40]. The convenience of this technique with children lies in its easiness to locate anatomical landmarks, early onset of anesthesia, and low pain perception during administration of the anesthetic solution [31]. It remains to be the gold standard technique [31]. IANB injections in the pediatric population has special consideration that differ from the adult population. Children’s anatomy varies significantly from adults impacting landmarks identification, needle placement and dosage. In 5-7year old children the mandibular foramen is located more inferiorly and posteriorly so needle insertion angulation will be almost at the level of the occlusal plane [32].

The Halstead technique for IANB has shown minimal tissue damage owing to its precise and contoured needle insertion [40]. There was a significantly lower level of pain upon injection with the double scalpel blade bevel needles in comparison with the conventional needle. This has been expressed both subjectively (FPS) and objectively (SEM score).

It is always recommended to select at least two pain scales when conducting behavioral research, especially in young children, considering their limited cognitive, emotional, and social development compared to adults. Therefore, in the present study both subjective and objective assessments were used [41]. Pain was measured subjectively by modifying the FPS from El ‘Maunuksela et al. [37, 42] to make it simpler to understand and interpret. This maximized the child’s response and reduced confusion. The SEM scale was used for objective assessment of pain as it has been frequently used in previous studies and has proved its accuracy to measure pain in children [43]. Different studies have previously investigated the effect of needle gauge and needle design on pain perception in patients, but the results were controversial [9, 44, 45]. McPherson et al. [1] in a randomized, double-blind split mouth clinical trial compared the subjective pain experience between large bore needles and standard bore needles both having an identical triple-bevel design. Several studies have also investigated needle bevel design on pain perception, but none were evaluated on pediatric patients.

The results of the present study are in accordance with Dau et al. [15] that revealed a significantly lower pain level when using a scalpel-designed bevel compared to triple and regular bevel designs. Moreover, Omoigui et al. [14] observed lower subjective pain levels when using low-angle bevel needles compared to the double bevel needle. In an in-vitro study carried out by Steele et al. [13] it was concluded that different bevel designs affect the insertion and withdrawal forces of the dental needle. This emphasizes the fact that double scalpel blade bevel design incises the tissues without tearing. This requires less force from the dental professional when injecting into dense tissues, contrary to what occurs with an IANB injection [20]. A high incidence of needle deformation was recorded in the current study in both needle groups, but the difference was not significant. This is in consistence with the results obtained by Rout et al. [46] that described needle deformation in 97.3% of cases. On the other hand, Dau et al. [15] observed that the scalpel bevel had a higher rate of deformation and a higher number of barbed hooks, with no effect on pain perception. This has been proposed as a major factor to be considered with IANB injections. Stacey and Hajjar [47] also reported 60% deformation of barbed needles. It has been demonstrated that a needle will barb when touching bone as in IANB injections. It has also been stated that a relationship exists between the barbing pattern and the placement of the needle bevel at the time of insertion. Hence, deformation could be linked to the technique of injection and operator variability. In the present study there was no favorite side for deviation. It has been stated that the needle is bent outward if the bevel is oriented medially and inward if the bevel faces the bone surface [15]. In the same context, needle deformation due to manufacturing has been evaluated as a pilot study in the present clinical trial. Ten unused needles of each type were tested. The results showed that one needle of each type had deformation on the tip before use due to an error in manufacturing.

To the best of our knowledge, this is the first study to evaluate the patented double scalpel blade bevel needles for IANB injection in children. The advantages of using this needle are that it does not need operator expertise, is easy to use and is cost-effective. There were no harms experienced in the test or control groups during intervention. No allergic reactions, trauma, or any adverse events were observed while working with the participants, nor reported afterwards.

A possible limitation of the present study was that the operator was not blinded because of the different types of needles primarily due to visible differences in needle design and appearance. Even though standardization was carried out by the operator, this limitation may introduce operator bias that may affect the performance and outcomes of injection. Future studies could explore the use of shielded or blinded needle devices to ensure blinding of the operation. Moreover, another blinded evaluator would have been beneficial to the study.

Another potential limitation was not evaluating the specific contribution of each pain phase during injection as penetration of the needle and deposition of the anesthetic to the overall pain perception, even though confounding variables were controlled. The observed pain relief was thus attributed to bevel design.

Within the limitation of this study and based on the previous data, the null hypothesis that there is no difference in pain perception and needle deformation between using a double scalpel blade bevel and a standard blade bevel needle during IANB anesthesia injection in children was partially rejected.

## Conclusion

For IANB anesthesia, the double scalpel blade bevel needle demonstrated significantly less injection pain. All dental needles demonstrated tip deformation after single use.

Further studies may be required to compare more types of needle designs regarding pain and deformation. A qualitative assessment correlating the degree of needle deformation and the pain experienced may also be an interesting aspect for evaluation. Furthermore, assessing pain at distinct phases of the injection process could be beneficial, as well as including uncooperative children in the study.

## Supplementary Information

Additional file 1

Additional file 2

Additional file 3

Additional file 4

## Data Availability

The data that support the findings of this study are not openly available due to reasons of sensitivity and are available from the corresponding author upon reasonable request.

## Abbreviations

IANB: Inferior alveolar nerve block
FPS: Face pain scale ‘modified Maunuksela’
SEM: Sensory, Eye, and Motor scale

## Declarations

### Trial registration

The trial was registered at ClinicalTrials.gov (NCT06242743) on the 5th February 2024, retrospective registration.
