# Supplementary material for: Effect of needle bevel type on pain perception in children during inferior alveolar nerve block anesthesia: randomized controlled clinical trial
Source: BMC Oral Health. 2025 Sep 2;25:1400. doi: 10.1186/s12903-025-06731-7 (PMC12406431; doi:10.1186/s12903-025-06731-7)
Supplement: Supplementary file 1 — Additional file 1 [file 12903_2025_6731_MOESM1_ESM.doc]

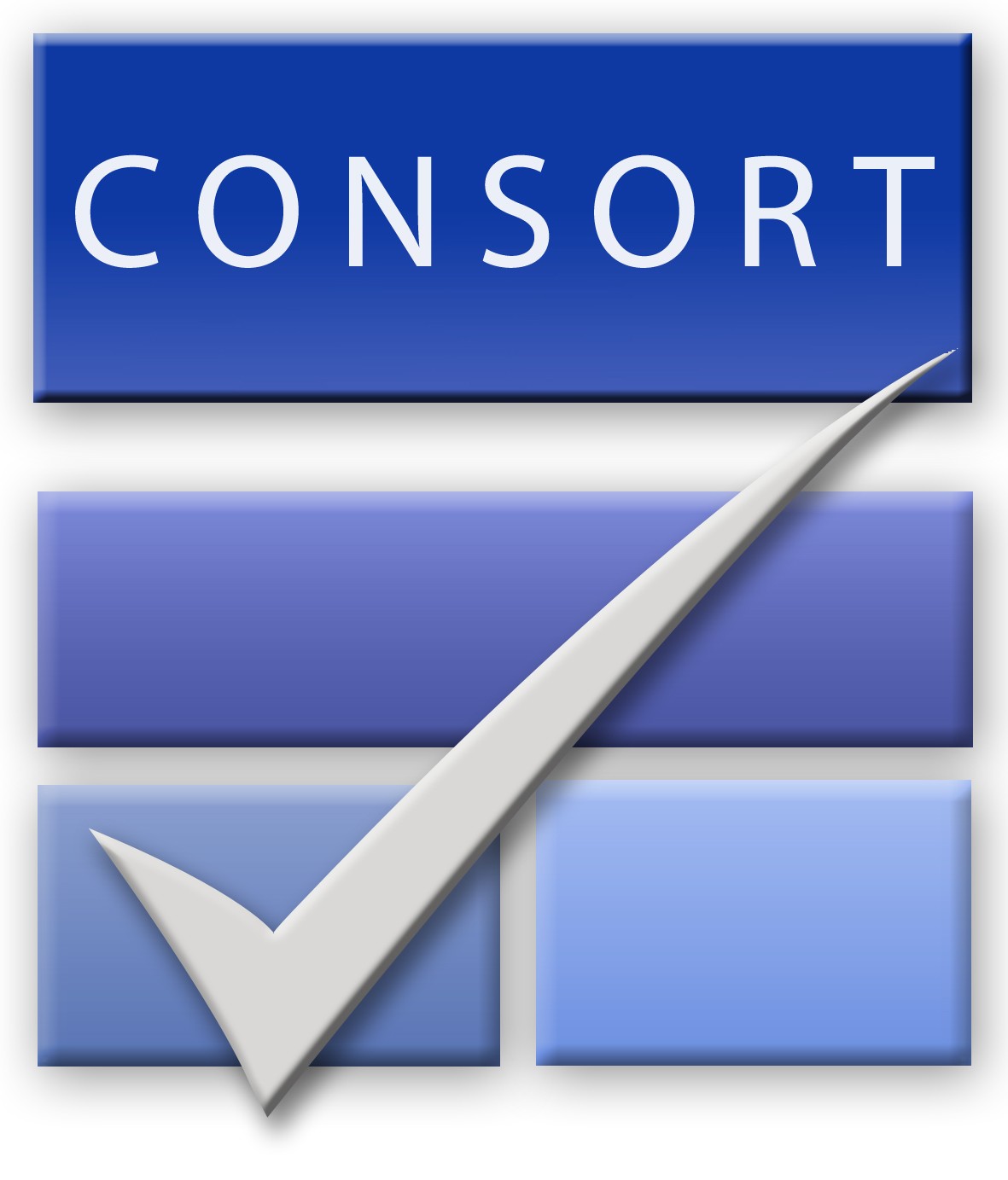
 CONSORT 2010 checklist of information to include when reporting a randomised trial*

| Section/Topic | Item No | Checklist item | Reported on page No |
| --- | --- | --- | --- |
| Title and abstract | | | |
|  | 1a | Identification as a randomised trial in the title | Page no 1 |
|  | 1b | Structured summary of trial design, methods, results, and conclusions (for specific guidance see CONSORT for abstracts) | Page no 2 |
| Introduction | | | |
| Background and objectives | 2a | Scientific background and explanation of rationale | Page no 3 |
|  | 2b | Specific objectives or hypotheses | Page no 4 line 4, 7 |
| Methods | | | |
| Trial design | 3a | Description of trial design (such as parallel, factorial) including allocation ratio | Page no 4 line 13 , 20 |
|  | 3b | Important changes to methods after trial commencement (such as eligibility criteria), with reasons | N/A |
| Participants | 4a | Eligibility criteria for participants | Page no 4 line 29 |
|  | 4b | Settings and locations where the data were collected | Page no 5 line1 |
| Interventions | 5 | The interventions for each group with sufficient details to allow replication, including how and when they were actually administered | Page no 6 line 3  Page no 4 line16 |
| Outcomes | 6a | Completely defined pre-specified primary and secondary outcome measures, including how and when they were assessed | Page no 6 line 24& page no 7 |
|  | 6b | Any changes to trial outcomes after the trial commenced, with reasons | N/A |
| Sample size | 7a | How sample size was determined | Page no 4 line21 |
|  | 7b | When applicable, explanation of any interim analyses and stopping guidelines | N/A |
| Randomisation: |  |  |  |
| Sequence generation | 8a | Method used to generate the random allocation sequence | Page no 5  line 14 |
|  | 8b | Type of randomisation; details of any restriction (such as blocking and block size) | Page no 5 line 12-14 |
| Allocation concealment mechanism | 9 | Mechanism used to implement the random allocation sequence (such as sequentially numbered containers), describing any steps taken to conceal the sequence until interventions were assigned | Page no 5  line 15 |
| Implementation | 10 | Who generated the random allocation sequence, who enrolled participants, and who assigned participants to interventions | Page no 5 line 17 |
| Blinding | 11a | If done, who was blinded after assignment to interventions (for example, participants, care providers, those assessing outcomes) and how | Page no 5 line 23 |
|  | 11b | If relevant, description of the similarity of interventions | Page no 6  line 8,15 |
| Statistical methods | 12a | Statistical methods used to compare groups for primary and secondary outcomes | Page no 7 line 22 |
|  | 12b | Methods for additional analyses, such as subgroup analyses and adjusted analyses | N/A |
| Results | | | |
| Participant flow (a diagram is strongly recommended) | 13a | For each group, the numbers of participants who were randomly assigned, received intended treatment, and were analysed for the primary outcome | Page no 8 line 2 |
|  | 13b | For each group, losses and exclusions after randomisation, together with reasons | Page no 8 ‘’fig 2’’ |
| Recruitment | 14a | Dates defining the periods of recruitment and follow-up | N/A |
|  | 14b | Why the trial ended or was stopped | N/A |
| Baseline data | 15 | A table showing baseline demographic and clinical characteristics for each group | Page no 8 line 5-8 |
| Numbers analysed | 16 | For each group, number of participants (denominator) included in each analysis and whether the analysis was by original assigned groups | Page no 8 |
| Outcomes and estimation | 17a | For each primary and secondary outcome, results for each group, and the estimated effect size and its precision (such as 95% confidence interval) | Page no 8 |
|  | 17b | For binary outcomes, presentation of both absolute and relative effect sizes is recommended | Page no 17  Table2,3 |
| Ancillary analyses | 18 | Results of any other analyses performed, including subgroup analyses and adjusted analyses, distinguishing pre-specified from exploratory | N/A |
| Harms | 19 | All important harms or unintended effects in each group (for specific guidance see CONSORT for harms) | Page no 3 line 24  Page no 10 line 14-16 |
| Discussion | | | |
| Limitations | 20 | Trial limitations, addressing sources of potential bias, imprecision, and, if relevant, multiplicity of analyses | Page no 10 line17-19 |
| Generalisability | 21 | Generalisability (external validity, applicability) of the trial findings | Page no 5 line1  Page no 6 line 3  Page no 6 line 24& page no 7  Page no 8 line 2 &fig 2  Page no 9 line 5 |
| Interpretation | 22 | Interpretation consistent with results, balancing benefits and harms, and considering other relevant evidence | Page no 9 |
| Other information | | |  |
| Registration | 23 | Registration number and name of trial registry | Page no 11 line 9 |
| Protocol | 24 | Where the full trial protocol can be accessed, if available | Page no 11 line 10 |
| Funding | 25 | Sources of funding and other support (such as supply of drugs), role of funders | N/A |

*We strongly recommend reading this statement in conjunction with the CONSORT 2010 Explanation and Elaboration for important clarifications on all the items. If relevant, we also recommend reading CONSORT extensions for cluster randomised trials, non-inferiority and equivalence trials, non-pharmacological treatments, herbal interventions, and pragmatic trials. Additional extensions are forthcoming: for those and for up to date references relevant to this checklist, see [www.consort-statement.org](http://www.consort-statement.org/).
