# Supplementary material for: Effect of needle bevel type on pain perception in children during inferior alveolar nerve block anesthesia: randomized controlled clinical trial
Source: BMC Oral Health. 2025 Sep 2;25:1400. doi: 10.1186/s12903-025-06731-7 (PMC12406431; doi:10.1186/s12903-025-06731-7)
Supplement: Supplementary file 2 — Additional file 2 [file 12903_2025_6731_MOESM2_ESM.docx]

**Baseline characteristics of the study groups**

|  | | Group I(test)  (n=33) | Group II(control)  (n=33) | *p* value |
| --- | --- | --- | --- | --- |
| Age: Mean ±SD | | 6.09 ±0.91 | 5.94 ±0.80 | 0.476 |
| Gender: n (%) | Males | 15 (45.5%) | 18 (54.5%) | 0.460 |
|  | Females | 18 (54.5%) | 15 (45.5%) |  |
