# Supplementary material for: Effect of needle bevel type on pain perception in children during inferior alveolar nerve block anesthesia: randomized controlled clinical trial
Source: BMC Oral Health. 2025 Sep 2;25:1400. doi: 10.1186/s12903-025-06731-7 (PMC12406431; doi:10.1186/s12903-025-06731-7)
Supplement: Supplementary file 3 — Additional file 3 [file 12903_2025_6731_MOESM3_ESM.docx]

Comparison of pain levels between groups among patients with deformed needles

| Pain scores | Group I  (n=28) | Group II  (n=28) | P value |
| --- | --- | --- | --- |
| Score A | 12 (42.9%) | 5 (17.9%) | 0.002* |
| Score B | 13 (46.4%) | 8 (28.6%) |  |
| Score C | 3 (10.7%) | 15 (53.6%) |  |
