# Supplementary figures and images for: Effect of needle bevel type on pain perception in children during inferior alveolar nerve block anesthesia: randomized controlled clinical trial
Source: BMC Oral Health. 2025 Sep 2;25:1400. doi: 10.1186/s12903-025-06731-7 (PMC12406431; doi:10.1186/s12903-025-06731-7)

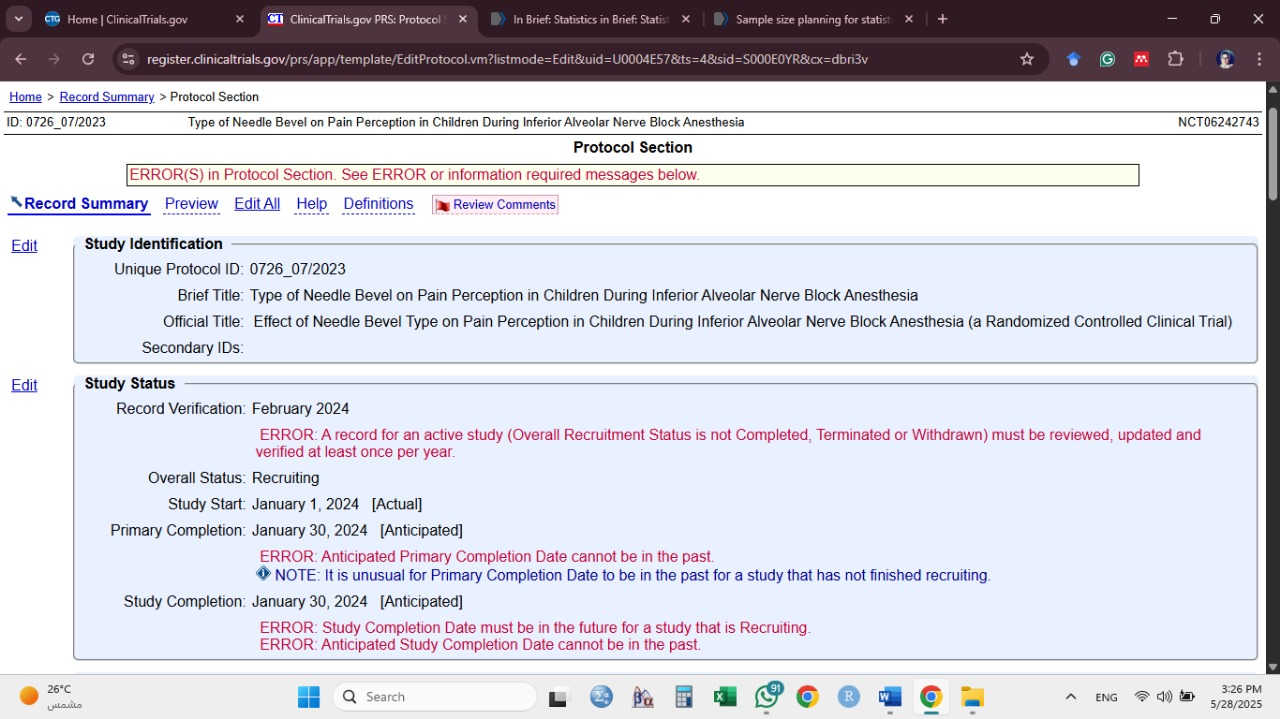

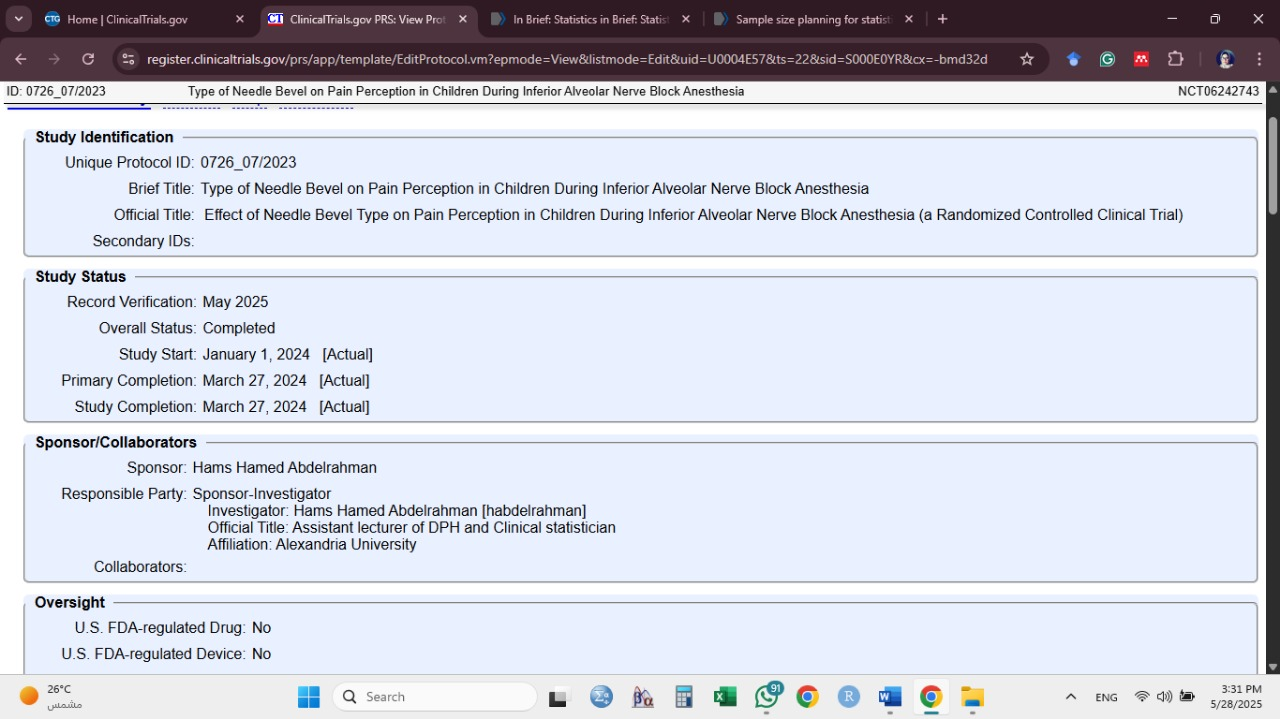

Supplement: Supplementary file 4 — Additional file 4 [file 12903_2025_6731_MOESM4_ESM.docx]
